# A randomized, active-controlled, multicentre clinical trial to evaluate the efficacy and safety of oral sitafloxacin versus levofloxacin in Chinese adults with acute uncomplicated or complicated urinary tract infection

**DOI:** 10.1080/07853890.2020.1861322

**Published:** 2020-12-17

**Authors:** Ying Li, Yousheng Yin, Xiaomei Peng, Hongguang Zheng, Fajun Fu, Zhenxiang Liu, Xiongfei Wu, Xiaoyan Wu, Song Zheng, Nan Chen, Leye He, Laicheng Ren, Zhaohui Ni, Detian Li, Peiyu Liang, Xiaoju Lv, Yingyuan Zhang

**Affiliations:** a Institute of Antibiotics, Huashan Hospital, Fudan University, Shanghai, China; b Key Laboratory of Clinical Pharmacology of Antibiotics, National Health and Family Planning Commission, Shanghai, China; c Department of Nephrology, The Affiliated Hospital of Guilin Medical College, Guilin, China; d Department of Urology, People’s Hospital of Guangxi Autonomous Region, Nanning, China; e Department of Nephrology, General Hospital of Northern Theater Command, Shenyang, China; f Department of Urology, Changsha Central Hospital, Changsha, China; g Department of Urology, Haikou People’s Hospital, Haikou, China; h Department of Nephrology, The First Hospital Affiliated to AMU (Southwest Hospital), Chongqing, China; i Department of Nephrology, Zhongnan Hospital, Wuhan University, Wuhan, China; j Department of Urology, Union Hospital Affiliated to Fujian Medical University, Fuzhou, China; k Department of Nephrology, Ruijin Hospital, Shanghai Jiao Tong University School of Medicine, Shanghai, China; l Department of Urology, The Third Xiangya Hospital of Central South University, Changsha, China; m Department of Urology, The Second Hospital of Shanxi Medical University, Taiyuan, China; n Department of Nephrology, Renji Hospital, Shanghai Jiao Tong University School of Medicine, Shanghai, China; o Department of Nephrology, Shengjing Hospital, China Medical University, Shenyang, China; p Department of Urology, The Affiliated Hospital of Hainan Medical University, Haikou, China; q Department of Infectious Diseases, West China Hospital, Sichuan University, Chengdu, China

**Keywords:** Sitafloxacin, levofloxacin, randomized controlled trial, urinary tract infection, efficacy, safety

## Abstract

**Purpose:**

To evaluate the efficacy and safety of oral sitafloxacin versus levofloxacin in Chinese adults with acute uncomplicated urinary tract infection (UTI) or complicated UTI.

**Methods:**

In this randomized, active-controlled clinical trial, the patients with acute uncomplicated UTI were randomized to receive sitafloxacin 100-mg once-daily (qd) or levofloxacin 500-mg qd orally for 3–5 days. The patients with complicated UTI were randomized to receive sitafloxacin 100-mg twice daily or levofloxacin 500-mg qd orally for 10–14 days. The primary endpoint was the clinical efficacy at test-of-cure (TOC) visit.

**Results:**

At TOC visit, the clinical cure rate was 89.2% (58/65) in sitafloxacin group and 97.1% (68/70) in levofloxacin group for the patients with acute uncomplicated UTI corresponding to the bacterial eradication rate of 97.1% (34/35) and 97.6% (41/42) (all *p* > .05), respectively. For the patients with complicated UTI, the clinical cure rate was 81.8% (27/33) in sitafloxacin group and 76.9% (20/26) in levofloxacin group corresponding to the bacterial eradication rate of 93.3% (14/15) and 63.6% (7/11) (all *p* > .05), respectively. Sitafloxacin and levofloxacin showed similar incidence of drug-related adverse events.

**Conclusions:**

Oral sitafloxacin is as effective and safe as levofloxacin in treating acute uncomplicated and complicated UTI.

**KEY MESSAGE:**
- Oral sitafloxacin showed similar clinical cure rate and bacterial eradication rate as levofloxacin for treatment of complicated and uncomplicated urinary tract infections (UTIs) in a randomized, active-controlled, multicentre clinical trial.
- Oral sitafloxacin is safe and well-tolerated in treating acute uncomplicated and complicated UTIs in Chinese adults.
- Sitafloxacin is a promising alternative treatment option for UTIs in adults.

## Introduction

Urinary tract infection (UTI) is one of the most common infectious diseases threatening normal daily living. The annual incidence of UTI is about 18/1000 persons [1]. Community-acquired uncomplicated UTI is the most common infection in females [2]. UTI is a prevalent and frequently encountered disease. Complicated UTI is recurrent and refractory to treatment. Hospital-acquired UTIs are usually associated with resistant microorganisms. Hence, UTIs have produced huge medical and economic burden on whole society. A study has shown that the medical and social cost due to work loss for caring UTIs sum up to $3.5 billion in the United States [3]. The urinary catheter-related infections in adults cost $896 per case on average [4]. UTI is also one of the most common infections in China.

The pathogens of UTI vary with the presence of complicating factors. However, the most frequently isolated pathogen is still *Escherichia coli*, which is the causative pathogen in about 80% of community acquired uncomplicated UTIs [5]. *E. coli* isolates are increasingly resistant to the established antimicrobial agents, including broad spectrum penicillins, cephalosporins, gentamicin and old fluoroquinolones. More than 30% of the urinary isolates of *E. coli* in Asian-Pacific region are resistant to third generation cephalosporins (cefotaxime, ceftriaxone and ceftazidime) and fourth generation cephalosporin (cefepime). About half of these *E. coli* strains are not susceptible (intermediate or resistant) to levofloxacin or ciprofloxacin [6]. Therefore, it is urgently needed at present time to have a new highly active antimicrobial agent available with broad coverage in clinical practice to fight UTIs, especially the complicated UTIs associated with resistant bacterial isolates.

Sitafloxacin is a fluoroquinolone antimicrobial agent developed by Daiichi Sankyo Company, Limited (Tokyo, Japan). It was approved and launched onto Japan market in 2008. Sitafloxacin is indicated for treatment of UTIs such as pyelonephritis and cystitis, and respiratory tract infections such as pneumonia, and the infections secondary to chronic respiratory diseases, as well as otitis media, nasal sinusitis, periodontitis, pericoronitis and oral infections [7]. Daiichi Sankyo (China) Holdings Co., Ltd. (Shanghai, China) sponsored this clinical study in Chinese adults with UTI for the purpose to facilitate and promote the clinical use of sitafloxacin in China. The result of this clinical study is reported as follows.

## Patients and methods

### Study design and patient population

This is a randomized, open-label, active-controlled multicentre phase 3 clinical trial designed to evaluate the efficacy and safety of sitafloxacin versus levofloxacin tablets in the treatment of Chinese adults with acute uncomplicated UTI or complicated UTI. This trial was registered at chinadrugtrials.org.cn (CTR20130047) and conducted in accordance with the ethical principles as laid down in the 1964 Declaration of Helsinki and its later amendments. The ethics committee at each site approved the study. Written informed consent was obtained from every patient before any study-related screening procedure started.

The patients were screened and enrolled from 34 study centres across China. The eligible subjects were stratified by disease (acute uncomplicated or complicated UTI) and randomized centrally by block randomization via an online interactive system. A unique subject identifier was assigned by study doctor to each patient. The containers were numbered sequentially to keep the sequence of random allocation. The patients with acute uncomplicated UTI were randomized in a ratio of 1:1 to receive sitafloxacin 100-mg once-daily (qd) or levofloxacin 500-mg qd orally for 3–5 consecutive days. The patients with complicated UTI were randomized in a ratio of 1:1 to receive sitafloxacin 100-mg twice daily (bid) or levofloxacin 500-mg qd orally for 10–14 consecutive days.

The patients were evaluated at the prespecified time points (visit): visit 1, within 48 hours before treatment to record baseline data; visit 2, 5–7 days after first dose (only for the patients with complicated UTI); visit 3, within two days after end of treatment (EOT visit) to evaluate the clinical efficacy and microbiological efficacy; visit 4, 5–9 days after EOT. Visit 4 was defined as test-of-cure (TOC) visit to evaluate clinical, microbiological and comprehensive efficacy.

#### Inclusion criteria

The enrolled patients met all the following inclusion criteria: 18–70 years of age (inclusive); did not receive antimicrobial therapy within 48 h before initiation of study drug; urinalysis ≥10 WBC/μL urine sample or ≥5 WBC per high power field (HPF); ≥10^5^ colony-forming units (CFU)/mL in the midstream urine culture within 48 h before treatment; urine pregnancy test was negative for women, and agreed to adopt effective contraceptive measures during study period until the last visit; voluntary to participate in this study and signed informed consent form.

The patients with acute uncomplicated UTI satisfied the following additional criteria: female; developed one or more clinical symptoms or signs of UTI (difficulty urinating, frequent urination, urinary urgency, painful urination/dysuria, lower abdominal pain) within 72 h before treatment; no anatomical anomaly and/or dysfunction in urinary tract. The patients with complicated UTI satisfied the following additional criteria: clinical diagnosis of complicated UTI who were expected to respond to oral antimicrobial treatment; experienced one or more clinical symptoms or signs of UTI (difficulty urinating, frequent urination, urinary urgency or painful urination/dysuria, nausea or vomiting, lower abdominal pain or flank pain, fever evidenced by oral temperature >37.5 °C or axillary temperature >37 °C, costovertebral angle tenderness or percussion pain) within 72 h before treatment; had underlying urinary tract disorder or one or more of the following conditions (diabetes mellitus, systemic lupus erythematosus, at least 100 mL residual urine after urination, neurogenic bladder, urinary obstruction due to nephrolithiasis, male, glomerular nephritis or nephrotic syndrome).

#### Exclusion criteria

The patients could not be enrolled if any one of the following was satisfied. Peripheral blood leucopenia (WBC <3.0 × 10^9^/L) or neutropenia (neutrophils <1.5 × 10^9^/L); immunocompromised due to immunosuppressants; probably to use other antimicrobial agents (except anaerobe-specific and antifungal agents); positive in urine fungal test; abnormal liver function test, aspartate transaminase (AST) and/or alanine transaminase (ALT) elevation >3 × upper limit of normal (ULN), and/or total bilirubin >2 × ULN; moderate or severe renal dysfunction evidenced by endogenous creatinine clearance rate <50 mL/min; cancer or other malignant disease; history of epilepsy or other central nervous disease; history of myasthenia gravis; prior QT elongation or serious heart disease; known or suspected hypersensitivity to sitafloxacin, levofloxacin or other fluoroquinolones; pregnant or lactating women; had participated in sitafloxacin clinical trial in the past; participated in the clinical study of other drugs at present or within 30 days before enrolment into this study; received any fluoroquinolone including levofloxacin within 1 week before enrolment; any other condition in the opinion of the investigator may increase the risk to patient or interfere with study results; the person directly involved in the activities of this study.

The patients with acute uncomplicated UTI could not be enrolled if any of the following was satisfied: acute uncomplicated UTI ≥3 episodes within one year or ≥2 episodes within half a year; immunocompromised due to use of glucocorticoids; diabetes mellitus. The patients with complicated UTI could not be enrolled if any of the following was satisfied: urinary diversion via enteric canal; indwelling urinary catheter; intermittent self-catheterization; concomitant with prostatitis or epididymitis; endogenous azotaemia due to kidney disease; proved diagnosis of sexually transmitted disease, which would not respond well to the study drugs; invasive procedures of urinary tract such as prostate biopsy within 30 days before treatment; use of glucocorticoids, the total dosage equivalent to prednisone daily dose ≥20-mg for longer than 2 weeks.

### Study endpoints

Efficacy evaluation included the evaluation of clinical efficacy, microbiological efficacy and comprehensive efficacy. The primary efficacy endpoint was defined as the clinical cure rate at TOC visit in per-protocol set (PPS).

#### Clinical efficacy evaluation

Clinical efficacy was evaluated as clinical cure or failure. Clinical cure was defined as the symptoms and signs of the target indication were resolved or recovered to the baseline state after treatment, and systemic antimicrobial therapy was no longer required for the target indication. Clinical failure was defined as the symptoms and signs of the target indication were persistent, or not resolved completely, or aggravated after treatment, or developed new symptom or sign of the index infection, and/or use another antimicrobial therapy to target the index infection. Clinical failure was also considered if the symptoms and/or signs were improved somewhat but still required to adjust or add treatment regimen.

#### Microbiological efficacy evaluation

Microbiological efficacy was evaluated as eradication, persistent, partially eradication, substitution, re-infection or colonization. The pathogenic isolates were tested by Kirby-Bauer disc method to determine their susceptibility to sitafloxacin and levofloxacin. Agar dilution method was used to determine the minimum inhibitory concentrations (MICs) of sitafloxacin, levofloxacin and other relevant antimicrobial agents against baseline bacterial isolates. The results of susceptibility testing were interpreted in accordance with the Clinical and Laboratory Standards Institute (CLSI) breakpoints in 2013.

#### Comprehensive efficacy evaluation

Comprehensive efficacy was evaluated as cure or failure. Cure was defined as clinical cure and microbiological eradication at TOC visit. Failure was defined as clinical failure and/or microbiological persistence at TOC visit. Comprehensive efficacy was evaluated only in patients with baseline pathogen, considering both clinical efficacy and microbiological efficacy.

### Safety evaluation

The subjects were observed closely to record their clinical adverse events (AEs) and laboratory abnormalities in details. The AEs were evaluated in accordance with corresponding criteria in terms of severity and relatedness to study drugs, including definitely, probably, possibly related, possibly or definitely unrelated. The AEs definitely, probably or possibly related to study drug were combined to calculate the incidence of adverse drug reactions.

The primary efficacy variable was the clinical efficacy rate at TOC visit in PPS for both the patients with acute uncomplicated UTI and those with complicated UTI. The secondary efficacy variables for the patients with acute uncomplicated UTI included the clinical efficacy rate at visit 3, microbiological efficacy at visit 3 and visit 4, and the comprehensive efficacy at visit 4, as well as pathogen-specific eradication rate at visit 3 and visit 4. The secondary efficacy variables for the patients with complicated UTI were the clinical efficacy rate at visit 3, microbiological efficacy at visit 3 and visit 4, and the comprehensive efficacy at visit 4, as well as pathogen-specific eradication rate at visit 3 and visit 4.

### Statistical analysis

SAS software 9.2 (SAS Inc., Cary, NC) was used to conduct statistical analysis. It was assumed that the clinical efficacy rate was higher than 90%, at least 100 patients were required to make the two-sided 95% confidence interval (CI) of clinical efficacy rate within the range of ±6%; hence, the total sample size in this study was 200 patients, including 140 patients with acute uncomplicated UTI (70 patients per group) and 60 patients with complicated UTI (30 patients per group).

The patients were defined as following analysis sets. Full analysis set (FAS) included all the randomized patients except those who had major protocol violation, did not take any study drug, had no post-randomization data, or did not have the target indication of this study. PPS was defined as all the patients in FAS who had clinical efficacy evaluation at TOC visit and did not have major violation of study protocol in terms of inclusion or exclusion criteria, concomitant medications or treatments, dosage (actual administered dose was 80–120% of the nominal dosage) or no data were available at visits after taking study drug. The discontinued patients were also included in PPS even if their clinical efficacy was “failure”. Microbiologically evaluable set (MES) was defined as all the patients in PPS who had baseline pathogenic isolate, and post-treatment follow-up data. Safety set (SS) included all the randomized patients who had received at least one dose of study drug. Descriptive statistics were presented for continuous variables at each time point, including number of patients, mean value, standard deviation, median, minimum and maximum value. The observed value at each time point and its change from baseline was also provided. Descriptive statistics including count and percentage were provided for categorical variables. The significance level was .05 for two-sided hypothesis test.

## Results

### Study population

**Figure 1.**
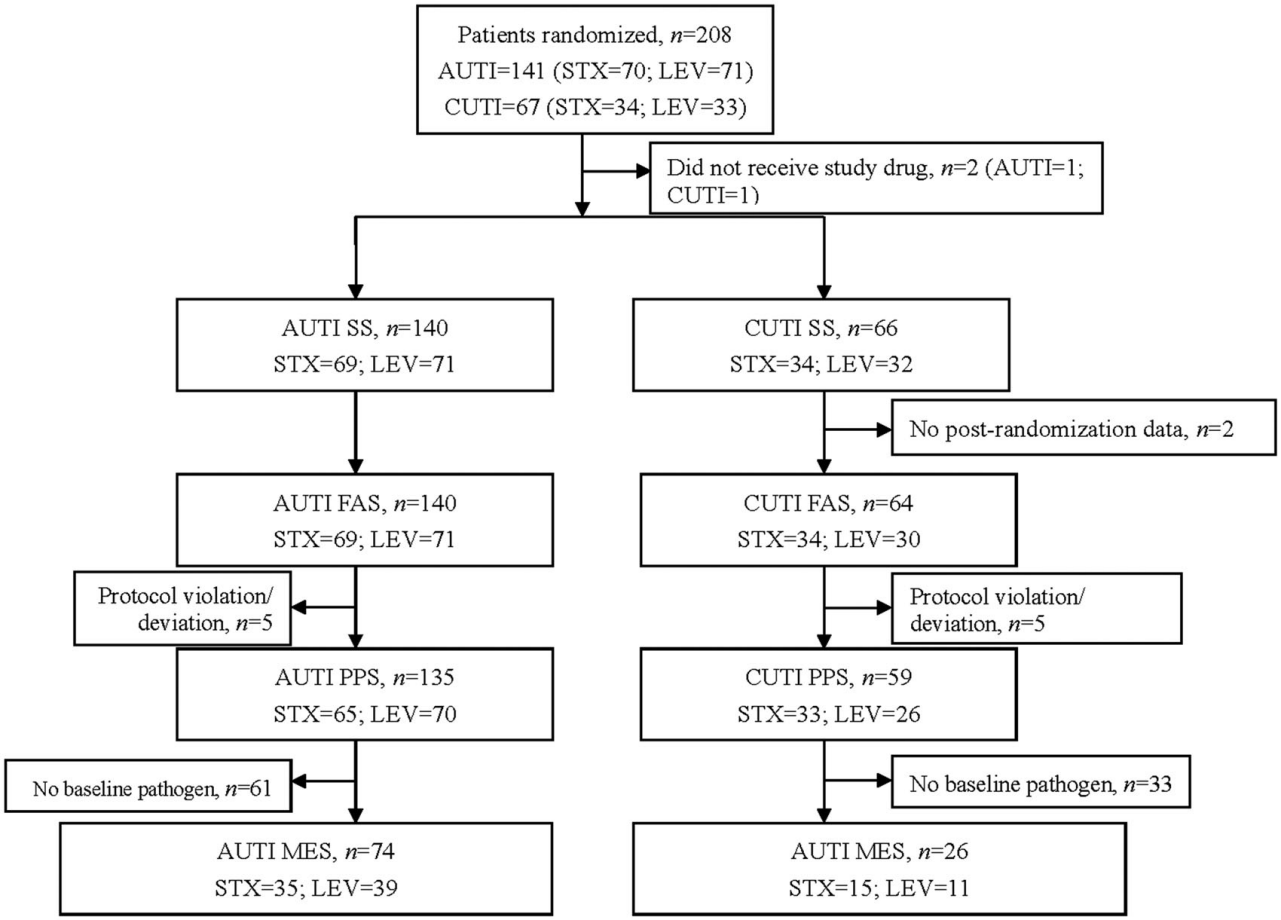
Study profile showing patient disposition. AUTI: acute uncomplicated urinary tract infection; CUTI: complicated urinary tract infection; FAS: full analysis set; LEV: levofloxacin; MES: microbiological evaluable set; PPS: per-protocol set; SS: safety set; STX: sitafloxacin.

A total of 208 patients were enrolled, 206 of which were included in SS. PPS included 135 patients with acute uncomplicated UTI (65 in sitafloxacin group, 70 in levofloxacin group) and 59 patients with complicated UTI (33 in sitafloxacin group, 26 in levofloxacin group). FAS included 140 patients with acute uncomplicated UTI and 64 patients with complicated UTI. MES included 74 cases of acute uncomplicated UTI and 26 cases of complicated UTI (Figure 1).

### Baseline characteristics of patients

#### Acute uncomplicated UTI

In FAS analysis, the patients were comparable between sitafloxacin group and levofloxacin group in terms of age, sex, body weight, height and body mass index. Underlying disease was found in low percentage of patients in both groups (Table 1). The common clinical symptoms and signs included lower urinary tract symptoms (frequent urination, urinary urgency, painful urination), urinary stuttering and lower abdominal discomfort/pain. The underlying diseases and UTI-related symptoms and signs were comparable at baseline between sitafloxacin group and levofloxacin group. The results in PPS were similar to FAS analysis.

**Table 1.** Demographic data and major underlying diseases of patients in two treatment groups – full analysis set.

Characteristics	Acute uncomplicated urinary tract infection		Complicated urinary tract infection	
	Sitafloxacin (*n* = 69)	Levofloxacin (*n* = 71)	Sitafloxacin (*n* = 34)	Levofloxacin (*n* = 30)
Age, years (mean ± SD)	40.6 ± 13.84	37.6 ± 13.67	47.9 ± 14.62	47.2 ± 14.60
Sex, males, *n* (%)	0	0	10 (29.4)	9 (30.0)
Body mass index, kg/m^2^ (mean ± SD)	21.69 ± 3.061	22.40 ± 3.161	23.70 ± 3.719	23.69 ± 3.737
Underlying disease, *n* (%)				
Nephrolithiasis	0	0	7 (20.6)	6 (20.0)
Glomerular nephritis	0	0	6 (17.6)	4 (13.4)
Diabetes mellitus	0	0	8 (23.5)	5 (16.7)
Hyperlipidaemia	1 (1.4)	0	3 (8.8)	2 (6.7)
Hypertension	2 (2.9)	4 (5.6)	8 (23.5)	6 (20.0)
Hydronephrosis	0	0	1 (2.9)	1 (3.3)
Nephrotic syndrome	0	0	0	1 (3.3)
Renal tubular acidosis	0	0	0	1 (3.3)

SD: standard deviation.

#### Complicated UTI

In FAS analysis, the patients were comparable between sitafloxacin group and levofloxacin group in terms of age, sex, body weight, height and body mass index. The main underlying diseases in FAS were hypertension, diabetes mellitus, nephrolithiasis and glomerular nephritis (Table 1). Febrile symptom was present in 8.8% (3/34) of the patients in sitafloxacin group and 23.3% (7/30) of the patients in levofloxacin group. The common clinical symptoms and signs included lower urinary tract symptoms (frequent urination, urinary urgency, painful urination), urinary stuttering, post micturition dribble, lower abdominal discomfort/pain, flank pain and costovertebral angle tenderness or percussion pain. The underlying diseases and UTI-related symptoms and signs were comparable at baseline between sitafloxacin group and levofloxacin group. The results in PPS were similar to FAS analysis.

### Efficacy results

#### Clinical efficacy

For acute uncomplicated UTI, the clinical cure rate was 86.8% in sitafloxacin group and 97.1% in levofloxacin group at visit 4 in FAS population. The clinical cure rate was 89.2% in sitafloxacin group and 97.1% in levofloxacin group at visit 4 in PPS population (*p* > .05) (Table 2).

**Table 2.** Comparison of clinical efficacy between sitafloxacin and levofloxacin.

Clinical cure rate	Sitafloxacin	Levofloxacin	Difference (95%CI)	*p* Value
	*n*/*N* (%)	*n*/*N* (%)		
Acute uncomplicated urinary tract infection				
Per-protocol set at test of cure visit	58/65 (89.2)	68/70 (97.1)	–7.9% (–16.4%, 0.6%)	.066
Full analysis set at test of cure visit	59/68 (86.8)	68/70 (97.1)	–10.4% (–19.3%, –1.4%)	.024
Complicated urinary tract infection				
Per-protocol set at test of cure visit	27/33 (81.8)	20/26 (76.9)	4.9% (–16.0%, 25.8%)	.643
Full analysis set at test of cure visit	28/34 (82.4)	20/27 (74.1)	8.3% (–12.6%, 29.2%)	.433

CI: confidence interval; *n*: number of patients clinically cured; *N*: total number of patients treated.

For complicated UTI, the clinical cure rate was 82.4% in sitafloxacin group and 74.1% in levofloxacin group at visit 4 in FAS population. The clinical cure rate was 81.8% in sitafloxacin group and 76.9% in levofloxacin group at visit 4 in PPS population (*p* > .05) (Table 2).

The clinical efficacy was also evaluated in terms of bacterial species. Sitafloxacin treatment resulted in a clinical cure rate of 92.3% (24/26) in the acute uncomplicated UTIs and 78.6% (11/14) in the complicated UTIs caused by *E. coli*. The corresponding clinical cure rate of levofloxacin was 96.7% (11/14) and 77.8% (7/9), respectively.

#### Microbiological efficacy

The microbiological eradication was analysed in MES population at both visit 3 and visit 4. For acute uncomplicated UTI, all the 26 strains of *E. coli* at baseline were eradicated after sitafloxacin treatment. The overall bacterial eradication rate was 97.1% (34/35) in sitafloxacin group. Levofloxacin eradicated 32 of the 33 baseline *E. coli* isolates. The overall bacterial eradication rate was 97.6% (41/42) in levofloxacin group (Table 3). In MES population, the microbiological success rate was 97.1% in sitafloxacin group and 97.4% in levofloxacin group. The per-patient microbiological success rate did not show significant difference between sitafloxacin group and levofloxacin group (*p* > .05) (Table 4).

**Table 3.** Microbial eradication rate in microbiologically evaluable patients at test of cure visit.

Pathogen	Acute uncomplicated urinary tract infection, % (*n*/*N*)		Complicated urinary tract infection, % (*n*/*N*)	
	Sitafloxacin (*N* = 35)	Levofloxacin (*N* = 39)	Sitafloxacin (*N* = 15)	Levofloxacin (*N* = 11)
*Escherichia coli*	100 (26/26)	97.0 (32/33)	92.9 (13/14)	66.7 (6/9)
Other gram-negative bacteria^a^	83.3 (5/6)	100 (5/5)	100 (1/1)	0 (0/1)
Gram-positive bacteria	100 (3/3)	100 (4/4)	NA	100 (1/1)

*n*: number of bacterial strains eradicated; *N*: total number of baseline isolates; NA: not available.

^a^ Including *Klebsiella pneumoniae* (6), *Proteus mirabilis* (2), *Enterobacter aerogenes* (1), *Citrobacter freundii* (1), *Aeromonas hydrophila* (2) and *Pseudomonas aeruginosa* (1).

**Table 4.** Microbiological efficacy in microbiologically evaluable set after treatment with sitafloxacin or levofloxacin.

	Acute uncomplicated urinary tract infection		Complicated urinary tract infection	
Time point	Sitafloxacin (*n* = 35)	Levofloxacin (*n* = 39)	Sitafloxacin (*n* = 15)	Levofloxacin (*n* = 11)
Visit 4, test of cure				
Bacterial eradication rate, % (95%CI)	97.1 (85.1, 99.9)	97.4 (86.5, 99.9)	93.3 (68.1, 99.8)	63.6 (30.8, 89.1)
Visit 3, end of treatment				
Bacterial eradication rate, % (95%CI)	94.3 (80.8, 99.3)	100 (91, 100)	93.3 (68.1, 99.8)	72.7 (39, 94)

CI: confidence interval.

For complicated UTI, sitafloxacin treatment eradicated 13 of the 14 baseline *E. coli* isolates. The overall bacterial eradication rate was 93.3% (14/15) in sitafloxacin group. Levofloxacin eradicated six of the nine baseline *E. coli* isolates. The overall bacterial eradication rate was 63.6% (7/11) in levofloxacin group (Table 3). In MES population, the microbiological success rate was 93.3% in sitafloxacin group and 63.6% in levofloxacin group. The per-patient microbiological success rate did not show significant difference between sitafloxacin group and levofloxacin group (*p* > .05) (Table 4).

The MICs of sitafloxacin and levofloxacin were determined against all baseline bacterial isolates. The MIC_90_ value of sitafloxacin and levofloxacin against 82 strains of *E. coli* was 1 mg/L and 8 mg/L, respectively. For the 12 strains of levofloxacin-resistant *E. coli*, the MIC of sitafloxacin was 1 mg/L against eight strains, and 2 mg/L against three strains. Sitafloxacin showed apparently lower MIC values than levofloxacin against gram-positive bacteria such as *Staphylococcus saprophyticus* and *Enterococcus faecalis* (Table 5).

**Table 5.** Minimum inhibitory concentrations of sitafloxacin and levofloxacin against major baseline pathogens.

Bacterial species (*n*)	Sitafloxacin, mg/L			Levofloxacin, mg/L		
	MIC range	MIC_50_	MIC_90_	MIC range	MIC_50_	MIC_90_
*Escherichia coli* (82)	≤0.06–8	≤0.06	1	≤0.06–32	0.25	8
Other *Enterobacteriaceae* species^a^ (10)	≤0.06–32	0.125	16	≤6.06 to >32	0.5	>32

MIC: minimum inhibitory concentration.

^a^ Including *Klebsiella pneumoniae* (6), *Proteus mirabilis* (2), *Enterobacter aerogenes* (1) and *Citrobacter freundii* (1).

#### Comprehensive efficacy

The comprehensive efficacy was evaluated in terms of the cure rate in MES population. For acute uncomplicated UTIs, the comprehensive cure rate was 88.6% (31/35, 95%CI: 73.3–96.8%) in sitafloxacin group, and 94.9% (37/39, 95%CI: 82.7–99.4%) in levofloxacin group. For complicated UTIs, the comprehensive cure rate was 80% (12/15, 95%CI: 51.9–95.7%) in sitafloxacin and 54.5% (6/11, 95%CI: 23.4–88.3%) in levofloxacin group.

### Safety results

A total of 206 patients were included in SS, including 140 patients with acute uncomplicated UTI (69 in sitafloxacin group and 71 in levofloxacin group) and 66 patients with complicated UTI (34 in sitafloxacin group and 32 in levofloxacin group).

As for the patients with acute uncomplicated UTI, the overall incidence of AEs was 36.2% (25/69) in sitafloxacin group and 21.1% (15/71) in levofloxacin group. The incidence of drug-related AEs was 27.5% (19/69) and 18.3% (13/71) respectively in the two groups. As for the patients with complicated UTI, the overall incidence of AEs was 35.3% (12/34) in sitafloxacin group and 40.6% (13/32) in levofloxacin group. The incidence of drug-related AEs was 26.5% (9/34) and 28.1% (9/32) respectively in the two groups.

For the patients with acute uncomplicated UTI specifically, sitafloxacin-related clinical AEs were found in 15.9% of the patients treated with sitafloxacin. The common clinical AEs were pruritus and palpitation. Sitafloxacin-related laboratory abnormalities were reported in 14.5% of the patients treated with sitafloxacin, mainly ALT elevation, AST elevation and WBC decreased. The incidence of levofloxacin-related clinical AEs was 16.9% in the patients treated with levofloxacin. The most common clinical AEs were dizziness and nausea, followed by headache. The incidence of levofloxacin-related laboratory abnormalities was 1.4% (one case of WBC decreased) (Table 6).

**Table 6.** Drug-related clinical adverse events and laboratory abnormalities occurring in at least 2% of the patients in any group based on safety set.

	Acute uncomplicated urinary tract infection		Complicated urinary tract infection	
	Sitafloxacin (*N* = 69)	Levofloxacin (*N* = 71)	Sitafloxacin (*N* = 34)	Levofloxacin (*N* = 32)
Adverse event	*n* (%)	*n* (%)	*n* (%)	*n* (%)
Clinical adverse event	11 (15.9)	12 (16.9)	4 (11.8)	6 (18.8)
Loss of appetite	0	1 (1.4)	0	2 (6.3)
Difficulty falling asleep	0	0	0	1 (3.1)
Headache	1(1.4)	3 (4.2)	1 (2.9)	0
Dizziness	1(1.4)	4 (5.6)	0	1 (3.1)
Palpitation	2 (2.9)	0	0	0
Abdominal distension	1 (1.4)	0	3 (8.8)	0
Nausea	1 (1.4)	4 (5.6)	0	2 (6.3)
Abdominal pain	0	0	0	2 (6.3)
Gastrointestinal disorder	0	0	0	1 (3.1)
Pruritus	3 (4.3)	0	0	0
Laboratory abnormality	10 (14.5)	1 (1.4)	5 (14.7)	3 (9.4)
Aspartic transaminase elevation	3 (4.3)	0	3 (8.8)	1 (3.1)
Alanine aminotransferase elevation	3 (4.3)	0	3 (8.8)	1 (3.1)
Elevated lactate dehydrogenase	2 (2.9)	0	2 (5.9)	1 (3.1)
WBC decreased	3 (4.3)	1 (1.4)	0	0
Neutrophils decreased	2 (2.9)	0	0	0
γ-Glutamyltransferase elevation	1 (1.4)	0	1 (2.9)	0
Elevated blood urea nitrogen	0	0	0	1 (3.1)

For the patients with complicated UTI specifically, sitafloxacin-related clinical AEs were found in 11.8% of the patients treated with sitafloxacin. The most common clinical AE was abdominal distension. Sitafloxacin-related laboratory abnormalities were reported in 14.7% of the patients treated with sitafloxacin, mainly ALT elevation and AST elevation. The incidence of levofloxacin-related clinical AEs was 18.8% in the patients treated with levofloxacin. The most common clinical AEs were loss of appetite, nausea and abdominal pain. The incidence of levofloxacin-related laboratory abnormalities was 9.4%, including ALT elevation, AST elevation, elevated lactate dehydrogenase and elevated blood urea nitrogen (one case each). No clinically significant electrocardiogram finding was reported in levofloxacin group (Table 6).

No SAE was reported in the patients with acute uncomplicated UTI or complicated UTI. A total of 29 cases of drug-related AEs were found in sitafloxacin group in the patients with acute uncomplicated UTI, all of which were mild in severity. A total of 20 cases of drug-related AEs were reported in levofloxacin group, 85.0% (17/20) of which were mild, while 15.0% (3/20) were moderate in severity. As for the patients with complicated UTI specifically, there were 13 cases of drug-related AEs in sitafloxacin group (all mild in severity) and 13 (all mild in severity) in levofloxacin group. There was no drug-related AE leading to treatment discontinuation.

## Discussion

Sitafloxacin is a broad-spectrum fluoroquinolone antimicrobial agent. It has shown good antimicrobial activity against gram-positive bacteria, gram-negative organisms and anaerobes, including fluoroquinolone-resistant strains [8]. The *in vitro* antimicrobial susceptibility testing with the bacterial isolates from UTIs and lower respiratory tract infections in Thailand showed that sitafloxacin was still active against ciprofloxacin-resistant gram-negative microorganisms, including multi-drug resistant gram-negative bacilli [9]. The PK/PD studies in healthy Chinese subjects indicate that sitafloxacin is absorbed rapidly after single-dose and multiple-dose oral administration of sitafloxacin tablets 100-mg q 12 h. The PK profile is linear within dose range from 50- to 200-mg. Most (more than 60%) of the administered dose was excreted from urine in unchanged form. The concentration of sitafloxacin in urine is at least fivefold higher than the MIC_90_ value of sitafloxacin against *E. coli* 8–12 hours post-dose [10]. A clinical study of sitafloxacin conducted in Thailand demonstrated that the *E. coli* (both ESBLs-positive and -negative strains) isolated from patients with complicated UTI or acute pyelonephritis were susceptible to sitafloxacin. Both bacterial eradication rate and clinical efficacy rate were higher than 95% in treating complicated UTI and acute pyelonephritis [11]. A randomized, double-blind, multicentre study in Japan showed that sitafloxacin 50-mg bid was noninferior to levofloxacin 100-mg three times a day in treating complicated UTIs (excluding catheter-related infection) in terms of clinical efficacy rate (sitafloxacin 96.1% versus levofloxacin 82.7%, *p* = .002). Bacterial eradication rate was 96.4% and 86.0%, respectively (*p* = .002) [12].

Sitafloxacin is similar to levofloxacin in terms of clinical and microbiological efficacy in treating acute uncomplicated UTIs. Sitafloxacin treatment is better than levofloxacin in terms of clinical cure rate and microbiological success rate in treating complicated UTIs, especially bacterial eradication rate, 93.3% (14/15) versus 63.6% (7/11). The difference between group is statistically insignificant, which may be due to small sample size. The above results are closely associated with the potent antimicrobial activity of sitafloxacin against *E. coli*, the major UTI pathogen. The pharmacodynamic study in China revealed that more than 70% of the *E. coli* strains resistant to conventional fluoroquinolones were still susceptible to sitafloxacin [10]. Of the pathogens in this study, 12 strains of *E. coli* were resistant to levofloxacin, but sitafloxacin was active against 11 of the 12 strains, evidenced by MIC 1 mg/L against eight strains and 2 mg/L against three strains. These results indicate that sitafloxacin is different from conventional fluoroquinolones, among which complete cross-resistance is observed, while sitafloxacin generally does not have cross-resistance with other conventional fluoroquinolones against most strains of *E. coli*, the predominant pathogen of UTIs. Sitafloxacin provides a new treatment option for clinical management of UTIs. It is expected to improve the outcome of patients with UTI, especially complicated UTI.

In the beginning of this century, the clinical studies of sitafloxacin in Japan have proved the overall good safety and tolerability of sitafloxacin. Combined safety analysis of clinical studies in Japanese subjects with respiratory tract infections, UTIs, ear, nose, throat infections, oral infections or reproductive system infections reported that of the 1220 patients, 409 (33.5%) developed AE (including laboratory abnormalities). The main AEs are diarrhoea, loose stool, headache, liver enzyme elevation and eosinophilia. In post-marketing clinical studies conducted in Japan, 148 (4.4%) of 3331 patients experienced AE (including laboratory abnormalities). The main AEs are diarrhoea, loose stool, skin rash and liver enzyme elevation [7]. Animal study indicated that phototoxicity of sitafloxacin could be milder than lomefloxacin and sparfloxacin in albino mice [13]. A randomized, controlled study demonstrated that sitafloxacin treatment (100-mg bid) was associated mild photosensitivity to ultraviolet ray in Caucasian population, which recovered to normal 24 h after treatment discontinuation, but sitafloxacin treatment (up to 200-mg bid) did not induce phototoxicity of clinical significance in Asian population [14]. Currently, there is no sitafloxacin-induced phototoxicity or photosensitivity reaction in Asian countries. The safety analysis of this study suggests that oral sitafloxacin 100-mg qd in treating acute uncomplicated UTI and oral sitafloxacin 100-mgbid in treating complicated UTI are as safe as levofloxacin. The drug-related clinical AEs and laboratory abnormalities were mostly mild in severity, transient and well-tolerated in this study. These results indicate that sitafloxacin is well-tolerated in treating patients with acute uncomplicated UTI or complicated UTI when administered by current dosing regimens.

The emerging antimicrobial resistance in the pathogens of UTIs poses a serious challenge to the efficacy of fluoroquinolone therapy [15]. Clinicians should pay careful attention to the susceptibility testing results of local bacterial isolates when prescribing sitafloxacin. In addition to sitafloxacin, some newer fluoroquinolones, such as finafloxacin and delafloxacin, are under development to address the drug-resistant pathogens. Sitafloxacin and these promising fluoroquinolones will provide more alternative treatment options for managing UTIs in adults [16–18].

## Conclusions

It is concluded from this study that the dosing regimen of sitafloxacin tablets 100-mg qd in acute uncomplicated UTI and sitafloxacin 100-mg bid in complicated UTI can provide good clinical and microbiological efficacy. The adverse reactions are infrequent, mild and transient. The proposed dosing regimen is sitafloxacin 100-mg qd, 3–5 days for treating acute uncomplicated UTIs and 100-mg bid, 10–14 days for treating complicated UTIs.

## Data Availability

The datasets generated in this study and the protocol are available from the corresponding author on reasonable request.
